# Racial disparities of pancreatic cancer in Georgia: a county‐wide comparison of incidence and mortality across the state, 2000–2011

**DOI:** 10.1002/cam4.552

**Published:** 2015-11-21

**Authors:** Lindsay Brotherton, Michael Welton, Sara W. Robb

**Affiliations:** ^1^ Department of Epidemiology and BiostatisticsCollege of Public HealthUniversity of GeorgiaAthensGeorgia

**Keywords:** Cluster analysis, pancreatic cancer, racial disparities

## Abstract

Understanding the geographic distribution of pancreatic cancer is important in assessing disease burden and identifying high‐risk populations. This study examined the geographic trends of pancreatic cancer incidence, mortality, and mortality‐to‐incidence ratios (MIRs) in Georgia, with a special focus on racial disparities of disease. Directly age‐adjusted pancreatic cancer incidence and mortality rates for Georgia counties (*N* = 159) were obtained for 2000–2011. Maps of county age‐adjusted disease rates and MIRs were generated separately for African Americans and Caucasians. Cluster analyses were conducted to identify unusual geographic aggregations of cancer cases or deaths. Pearson correlation coefficients were calculated to examine associations between county health factors (e.g., health behaviors, clinical care, and physical environment) and pancreatic cancer incidence or mortality rates. African Americans displayed a significantly higher age‐adjusted incidence (14.6/100,000) and mortality rate (13.3/100,000), compared to Caucasians. Cluster analyses identified five significant incidence clusters and four significant mortality clusters among Caucasians; one significant incidence cluster and two significant mortality clusters were identified among African Americans. Weak but significant correlations were noted between physical environment and pancreatic cancer incidence (*ρ *= 0.16, *P* = 0.04) and mortality (*ρ *= 0.18, *P* = 0.02) among African Americans. A disproportion burden of pancreatic cancer incidence and mortality was exhibited among African Americans in Georgia. Disease intervention efforts should be implemented in high‐risk areas, such as the southwest and central region of the state. Future studies should assess health behaviors and physical environment in relationship with the spatial distribution of pancreatic cancer.

## Introduction

Pancreatic adenocarcinoma, commonly known as pancreatic cancer, represents the 12th most common type of cancer in the United States 1. With a national age‐adjusted incidence rate of 12.3 per 100,000, approximately 46,420 individuals were diagnosed with pancreatic cancer in 2014 1. Although the disease is relatively rare, pancreatic cancer is one of the most fatal cancers among adults in the United States. Pancreatic cancer has the lowest 5‐year survival rate of any cancer, is the fourth leading cause of cancer death in the nation, and approximates breast cancer's death toll 1.

No major professional group recommends routine screening for pancreatic cancer; the natural history of the disease is not fully understood and current screening tools, including imaging modalities and serum biomarkers, are limited in diagnostic accuracy 2. Because screening is not recommended and the cancer typically develops with few symptoms, the majority of patients are diagnosed at an advanced stage. As an aggressive disease, the 5‐year survival rate is <5% 3, and despite occasional cases of early disease detection, nearly all patients die from pancreatic cancer within 1–2 years 4.

The main risk factors of pancreatic cancer include smoking, obesity, long‐standing diabetes, and family history of disease 5. Cigarette smoking is the most well‐established risk factor for pancreatic cancer 6, 7, 8, 9. Smoking cigarettes causes a 75% increase in the risk of pancreatic cancer compared to nonsmokers 10. Accordingly, 20% of pancreatic tumors may be attributed to cigarette smoking 10. Epidemiological investigations have also reported a 20–50% increased risk of disease among obese relative to nonobese individuals 11. Although the relationship between diabetes and pancreatic cancer is complex, long‐term type 2 diabetes has been associated with a significant increase in the risk of pancreatic cancer as well 12. Lastly, up to 10% of patients have a family history of pancreatic cancer 13, and the risk of disease is considerably greater (80%) among persons with affected family members compared to those without 14.

As a disease of notable mortality, further research is greatly warranted. Understanding the geographic distribution of pancreatic cancer is important in assessing disease burden and identifying high‐risk populations. To date, very few studies have examined pancreatic cancer at the state level. Although previous investigations have examined pancreatic cancer clustering in the state of Florida 15, Texas 16, and Massachusetts 17, none have looked at disease clustering by racial subgroups.

Racial disparities in healthcare and outcomes have been evident for almost all cancer sites 1. These disparities persist through each step of the cancer‐care continuum, ranging from the unequal distribution of cancer risk factors to inequities in early diagnosis and appropriate treatment 18, 19. African Americans display the highest death rate and shortest survival of any racial group for most cancers in the United States 20. The increased mortality within this race may be attributed to more aggressive cancers and more advanced stage at diagnosis as well as differences in treatment, socioeconomic factors, physician characteristics, and personal beliefs 21. In terms of pancreatic cancer in the United States, African Americans have been diagnosed with disease at a 48% higher rate compared to Caucasians 22, and have the poorest median survival time compared to other races 23.

The state of Georgia is a fitting candidate for the assessment of racial and geographic disparities of disease. While Georgia's pancreatic cancer incidence rate is slightly below the national rate (11.6 vs. 12.2) 24, the state has a relatively substantial African American population; approximately 31% of Georgia's population is African American 25. Additionally, the state age‐adjusted pancreatic cancer incidence rates for African Americans and Caucasians is 10.8 and 14.6, respectively, which are suggestive of potential disease disparities.

The objectives of this paper were as follows: (1) to identify pancreatic cancer racial disparities, including incidence, mortality, and mortality‐to‐incidence ratios in Georgia; (2) to illustrate the geographic distribution of pancreatic cancer within the state of Georgia; and to (3) explore health factors associated with pancreatic cancer at the county level. Ultimately, a better understanding of the geographic distribution of disease by race will assist in efforts to both manage current disease and prevent future disease in Georgia.

## Methods

### Data sources

This investigation evaluated age‐adjusted incidence and mortality rates at the county level stratified by race (African American vs. Caucasian vs. all races combined). The mortality‐to‐incidence ratio (MIR), which is calculated as the age‐adjusted mortality rate divided by the age‐adjusted incidence rate, is a measure of mortality risk that adjusts for underlying differences in incidence 26. The MIR has previously been used to describe cancer in Georgia for other cancer sites 26 and serves as a population‐based measure of fatality (1/survival), given incidence 27. Age‐adjusted (2000 US Standard Population) incidence rates, mortality rates, and MIRs for Georgia counties (*N* = 159) were generated for the years 2000–2011.

Cancer data for this study were obtained in January 2015 from the Surveillance, Epidemiology and End Results (SEER) Program of the National Cancer Institute, SEER*Stat (version 8.1.5) 28. SEER*Stat was used to calculate age‐adjusted disease rates by county and race. County data including the county population, number of pancreatic cancer cases, and number of pancreatic cancer deaths were also obtained from SEER*Stat 28.

County health data were obtained from *County Health Rankings & Roadmaps* 29. *County Health Rankings & Roadmaps* synthesizes health information from a variety of national data sources including The Behavioral Risk Factor Surveillance System, The National Center for Health Statistics, and The United States Census Bureau 29. County health factors examined within this study included *health behaviors*, a composite measure of tobacco use, diet and exercise, alcohol use, motor vehicle crash death rate, and unsafe sex; *social and economic factors*, a composite measure of education, employment, income, family and social support, and community safety; *physical environment*, a composite measure of environmental quality and built environment; and *clinical care*, a composite measure of access to care and quality of care 29. County‐level *Z*‐scores (i.e., standardized measures of each health measure relative to other Georgia counties) for each health factor were obtained for the year 2011. A positive *Z*‐score indicated a value higher (i.e., “greater risk” for worse health outcomes) than the average of all Georgia counties; a negative *Z*‐score indicated a value lower (i.e., “lower risk” for worse health outcomes) than the average of all Georgia counties.

### Data analysis

The state age‐adjusted incidence and mortality rates of pancreatic cancer, along with MIRs, were generated and stratified by race and gender. Corresponding 95% confidence intervals (CI) were reported. Special methods previously used for MIRs were implemented to calculate 95% CIs using *F* intervals 30. For both races, tumor staging and age at diagnosis were examined for the 2000–2011 time period. Tumor staging was classified as localized, regional, distant, or unknown.

The geographic distribution of pancreatic cancer incidence and mortality was analyzed for clustering among the 159 counties in Georgia. Age‐adjusted incidence and mortality rates, and MIRs of Georgia counties were mapped for Caucasians, African Americans, and the total Georgia population during the years 2000–2011. Map generation was performed in R version 3.1.2 31. Prior to identifying disease clusters and calculating Pearson correlation coefficients, county outliers were identified based on studentized residuals above *r* = 2.50, and normality of the variables was confirmed using a Shapiro–Wilk Normality test. County outliers were excluded from subsequent cluster and correlation analyses.

Cluster analyses were conducted to identify areas within the state that exhibited a significantly greater than expected number of pancreatic cancer cases or deaths. Cluster analyses were performed using the software program SaTScan version 9.3.1 32. A Poisson‐based model was evaluated where the number of events in a geographic area was Poisson‐distributed, according to the known underlying population at risk. For all models, SaTScan used a purely spatial circular scan statistic, 3% spatial scanning window, and 999 simulations 32. Cluster analyses were performed for all races combined, and then stratified by racial subgroups (African Americans vs. Caucasians). Primary and secondary clusters were identified and presented. Primary clusters were considered significant at *P* ≤ 0.05. Secondary clusters were evaluated more conservatively because of SatScan's program inability to adjust for experiment wide error in secondary clusters. Secondary clusters were considered significant at *P* ≤ 0.01. Clusters were displayed on maps illustrating age‐adjusted county incidence and mortality rates. Details on each significant cluster, including cluster center (county), radius, *P*‐value, and relative risk were also presented.

For each health factor, correlations of *Z*‐scores to age‐adjusted pancreatic cancer incidence and mortality rates at the county level were calculated. Pearson correlation coefficients and one‐sided *P* values were calculated for pancreatic cancer by race. Correlations were considered statistically significant at *P* < 0.05.

## Results

From 2000 to 2011, there were 7651 pancreatic cancer cases among Caucasians and 3082 cases among African Americans in Georgia. For both African Americans and Caucasians, the majority of tumors were regional (24 and 27%) or distant (54 and 48%) at the time of diagnosis. In terms of age at diagnosis, Caucasians were generally diagnosed between the ages of 60–79 (54%) or 80 and older (23%), whereas African Americans were commonly diagnosed between the ages of 60–79 (51%) or 40–59 (30%).

**Figure 1:**
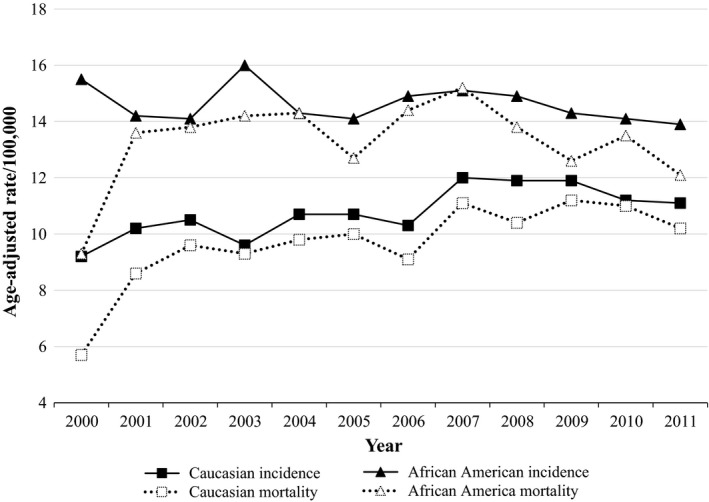
Georgia age‐adjusted pancreatic cancer rates by time and race, 2000–2011. Rates are per 100,000 and age‐adjusted to the 2000 US Standard Population. Data Source: SEER*Stat (version 8.1.5).

Over the study period, the state age‐adjusted incidence and mortality rate of pancreatic cancer per 100,000 was 11.6 and 10.5, respectively. African Americans had an overall significantly higher age‐adjusted incidence rate (14.6/100,000) and mortality rate (13.3/100,000) compared to Caucasians (10.8/100,000 and 9.7/100,000, respectively). Annual mortality and incidence rates by race are displayed in Figure 1. All annual disease rates for African Americans were significantly greater than annual disease rates for Caucasians, with the exception of rates in 2009 and 2011; African Americans had higher disease rates in 2009 and 2011, but these results were not statistically significant compared to Caucasian rates. In terms of temporal trends, annual disease rates for Caucasians gradually increased until 2007, after which rates appeared to stabilize. For African Americans, annual incidence rates were fairly stable during the study duration, while annual mortality rates gradually increased until 2007 and then began declining (Fig. 1).

Disease rates by gender are presented in Table 1. Males had significantly higher age‐adjusted mortality and incidence rates compared to females, among both races. For our study population, African American males had the highest incidence rate (16.5/100,000) as well as the highest mortality rate (15.2/100,000). Female Caucasians had the lowest incidence rate (9.3/100,000) as well as the lowest mortality rate (8.4/100,000). MIRs were not significantly different between the different strata; all MIR values were noticeably high compared to other cancers 1, ranging from 0.90 to 0.92, which indicates very low disease survival (Table 1).

**Table 1:** Incidence, mortality, and mortality‐to‐incidence ratio (MIR) by race and sex in Georgia, 2000–2011

Race	Sex	Incidence rate^a^ (95% CI)	Mortality rate^a^ (95% CI)	MIR (95% CI)^b^
All^c^	Male	13.4 (13.0–13.7)	12.2 (11.8–12.6)	0.91 (0.876–0.952)
	Female	10.2 (9.9–10.5)	9.1 (8.9–9.4)	0.90 (0.862–0.932)
African American	Male	16.5 (15.5–17.4)	15.2 (14.3–16.2)	0.92 (0.849–1.00)
	Female	13.2 (12.5–13.8)	11.9 (11.3–12.6)	0.91 (0.842–0.974)
Caucasian	Male	12.7 (12.3–13.1)	11.5 (11.1–11.9)	0.91 (0.868–0.956)
	Female	9.3 (9.0–9.6)	8.4 (8.1–8.6)	0.90 (0.853–0.938)

MIR, mortality‐to‐incidence ratio; CI, confidence interval; Data source, SEER*Stat (version 8.1.5).

a Rates are per 100,000 and directly age‐adjusted to the 2000 US Standard Population.

b 95% CI for MIRs were calculated using the F‐method 32.

c Includes Caucasian, African American, American Indian/Alaska native, Asian or Pacific Islander, and unknown race.

Lincoln (studentized residual = 3.83) and Treutlen (studentized residual = 3.72) counties were identified as incidence outliers and excluded from the cluster analysis and the correlation analysis examining pancreatic cancer incidence in Georgia. Age‐adjusted pancreatic cancer incidence rates were normally distributed (Shapiro–Wilk = 0.99; *P* = 0.14). When examining mortality rates, Lincoln (studentized residual = 3.73), Treutlen (studentized residual = 3.94), Heard (studentized residual = 3.37), and Wilkinson (studentized residual = 2.63) counties were identified as outliers and excluded from the cluster analysis and the correlation analysis examining pancreatic cancer mortality in Georgia. Age‐adjusted pancreatic cancer mortality rates were normally distributed (Shapiro–Wilk = 0.99; *P* = 0.72).

**Figure 2:**
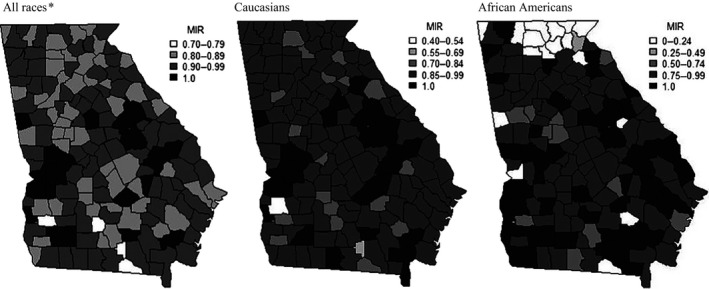
Pancreatic cancer mortality‐to‐incidence ratios (MIRs) by race and county, 2000–2011. Rates used to calculate MIRs are per 100,000 and age‐adjusted to the 2000 US Standard Population. Data Source: SEER*Stat (version 8.1.5). *Includes Caucasian, African American, American Indian/Alaska native, Asian or Pacific Islander, and unknown race.

Figure 2 displays maps of MIRs by race and county. When examining county MIRs for all races combined, ratios ranged from 0.75 to 1.0, and approximately 70% of all Georgia counties had MIR values >0.89. When examining MIRs by racial subgroups, ratios remained consistently high for both African Americans and Caucasians, with the exception of African American MIRs in the northern region of the state; many counties (*n* = 12) in this region displayed very low population percentages of African Americans and had no reported pancreatic cancer incidence cases or deaths among African Americans. Consequently, MIRs for these African Americans in these counties were not calculated.

**Figure 3:**
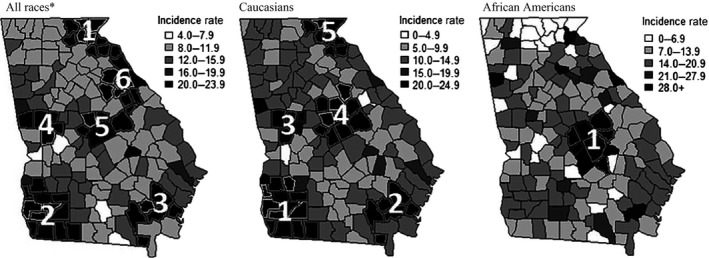
Age‐adjusted pancreatic cancer incidence rates and clusters of pancreatic cancer incidence cases by race and county, 2000–2011. *Note*: Pancreatic cancer incidence clusters are numbered and indicated by black county shading. Numbers correspond to the cluster numbers in Table 2. Further details on each cluster can be found in Table 2. *Note*: Primary clusters are considered significant at *P* ≤ 0.05. Secondary clusters are considered significant at *P* ≤ 0.01. SaTScan Parameters: Discrete Poisson model, 3% Spatial Scanning Window, 999 simulations. Data Source: SEER*Stat (version 8.1.5). Rates are per 100,000 and age‐adjusted to the 2000 US Standard Population. *Includes Caucasian, African American, American Indian/Alaska native, Asian or Pacific Islander, and unknown race.

Pancreatic cancer incidence rates, examined geographically by county and race, are displayed in Figure 3. Among the separate race categories, the highest incidence rates were observed among African Americans in southern Georgia in Brantley County (70.5/100,000), and in northern Georgia in Haralson County (38.9/100,000) and Lincoln County (35.2/100,000). Among Caucasians, Quitman County, found in the southern region of the state, and Heard County, found in the central region of the state, had notably high pancreatic cancer incidence, with age‐adjusted rates of 23.4/100,000 and 20.1/100,000, respectively.

Cluster analyses identified six incidence clusters, in which approximately 18% of all pancreatic cancer cases were located during the study period. The largest clusters were detected in South Georgia, centered in Seminole County (cluster no. 2; 94 km radius; *P* < 0.0001; Fig. 3; Table 2) and North Georgia centered in Rabun County (cluster no. 1; 71 km radius; *P* < 0.0001; Fig. 3; Table 2). There were five incidence clusters identified within the Caucasian population and one incidence cluster identified within the African American population. The largest cluster in the Caucasian population was centered in Seminole County (cluster no. 1; 101 km radius; *P* < 0.0001; Fig. 3; Table 2) and the single cluster identified for African Americans was centered in Laurens County (cluster no. 1; 52 km radius; *P* = 0.003; Fig. 3; Table 2).

**Table 2:** Statistically significant clusters^b^ of county‐level pancreatic cancer incidence and mortality by race in Georgia, 2000–2011

Cluster number^c^	Cluster center county^d^	Radius (km)	*P*‐value^a^	O/E	Relative risk^e^
Incidence clusters					
All races combined^f^					
1	Rabun	70.86	1.5e‐13	412/271	1.55
2	Seminole	94.26	*2.3e‐8*	435/315	1.40
3	Brantley	45.98	*3.0e‐9*	311/207	1.52
4	Meriwether	35.02	*9.1e‐7*	244/164	1.50
5	Wilkinson	45.41	*1.5e‐5*	422/322	1.33
6	Wilkes	59.24	*0.0026*	165/113	1.47
Caucasian					
1	Seminole	100.54	*1.0e‐9*	267/171	1.59
2	Brantley	45.98	*8.2e‐7*	250/169	1.50
3	Upson	34.39	*0.0001*	126/78	1.63
4	Baldwin	53.76	*0.0057*	289/225	1.30
5	Rabun	59.35	*1.2e‐8*	310/210	1.50
African American					
1	Laurens	51.53	*0.0028*	97/61	1.64
Mortality clusters					
All races combined^f^					
1	Rabun	70.86	*8.7e‐13*	370/241	1.57
2	Seminole	94.26	*1.6e‐9*	400/279	1.45
3	Brantley	45.98	*7.7e‐10*	285/184	1.57
4	Meriwether	35.02	*5.2e‐6*	217/145	1.51
5	Monroe	39.21	*9.7e‐6*	385/288	1.35
6	Washington	68.23	*0.00047*	339/260	1.32
Caucasian					
1	Grady	79.94	*4.1e‐9*	300/198	1.54
2	Brantley	45.98	*4.2e‐7*	230/151	1.55
3	Monroe	41.55	*0.00018*	276/202	1.38
4	Rabun	59.30	*3.9e‐8*	258/169	1.55
African American					
1	Wilkes	42.85	*0.015*	53/29	1.82
2	Decatur	82.97	*0.010*	110/73	1.54

**Figure 4:**
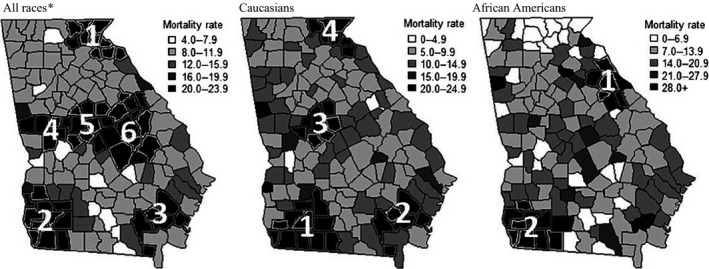
Age‐adjusted pancreatic cancer mortality rates and clusters of pancreatic cancer mortality cases by race and county, 2000–2011. *Note*: Pancreatic cancer mortality clusters are numbered and indicated by black county shading. Numbers correspond to cluster numbers in Table 2. Further details on each cluster can be found in Table 2. *Note*: Primary clusters are considered significant at *P* ≤ 0.05. Secondary clusters are considered significant at *P* ≤ 0.01. SaTScan Parameters: Discrete Poisson model, 3% Spatial Scanning Window, 999 simulations. Data Source: SEER*Stat (version 8.1.5). Rates are per 100,000 and age‐adjusted to the 2000 US Standard Population. *Includes Caucasian, African American, American Indian/Alaska native, Asian or Pacific Islander, and unknown race.

O/E, observed/expected. Italics indicate significant cluster.

a Primary clusters are considered significant *P* ≤ 0.05. Secondary clusters are considered significant at *P* ≤ 0.01.

b Purely spatial circular scan statistic was used with 3% spatial scanning window and 999 simulations.

c Cluster numbers correspond to figure 3 (incidence) and figure 4 (mortality).

d The county in which the cluster's geographic centroid falls inside.

e Relative risk is the estimated risk within the cluster divided by the estimated risk outside the cluster.

f Includes Caucasian, African American, American Indian/Alaska native, Asian or Pacific Islander, and unknown race.

Pancreatic cancer mortality rates examined geographically by county and race, are shown in Figure 4. Among separate race categories, the highest mortality rates were observed for African Americans in Brantley County (70.5/100,000), Haralson County (35.3/100,000), and Lincoln County (35.4/100,000). Among Caucasians, Quitman County and Heard County also had particularly high pancreatic cancer mortality, with age‐adjusted rates of 21.2/100,000 and 20.3/100,000, respectively.

Cluster analyses identified six clusters in which approximately 21% of all pancreatic cancer deaths were located during the study period. The largest clusters were detected in South Georgia, centered in Seminole county (cluster no. 2; 94 km radius; *P* < 0.0001; Fig. 4; Table 2) and North Georgia centered in Rabun county (cluster no. 1; 71 km radius; *P* < 0.0001; Fig. 4; Table 2). There were four mortality clusters identified within the Caucasian population and two mortality cluster identified within the African American population. The largest cluster in the Caucasian population was centered in Grady County (cluster no. 1; 80 km radius; *P* < 0.0001; Fig. 4; Table 2) and the largest cluster for African Americans was centered in Decatur County (cluster no. 2; 83 km radius; *P* = 0.010; Fig. 4; Table 2).

Table 3 displays the correlations between county incidence and mortality rates and health factor groupings. Expectations were that higher *Z*‐scores (i.e., counties at a “higher risk” for worse health outcomes) would be correlated positively with higher age‐adjusted incidence and mortality rates. A weak but significant positive correlation was detected between physical environment and pancreatic cancer incidence in African Americans (*ρ *= 0.16, *P* = 0.04). An additional weak positive correlation was detected between physical environment and pancreatic cancer mortality in African Americans (*ρ *= 0.18, *P* = 0.02). Although the associations were not statistically significant, weak positive associations in African Americans between health behaviors and pancreatic cancer incidence rate (*ρ *= 0.13, *P* = 0.10) and health behaviors and mortality rates (*ρ *= 0.14, *P* = 0.09) were suggested. All remaining correlations were insignificant.

**Table 3:** Correlation coefficients between county health factors and age‐adjusted pancreatic cancer incidence and mortality rates by race, 2000–2011 (*N* = 156)^a^

	All races^b^		African American		Caucasian	
	*ρ*	*P*	*ρ*	*P*	*ρ*	*P*
Health behaviors						
Incidence rate^c^	0.083	0.31	0.132	0.10	0.057	0.49
Mortality rate^d^	0.098	0.23	0.137	0.09	0.048	0.56
Social and economic factors						
Incidence rate^c^	0.023	0.78	0.071	0.38	0.105	0.20
Mortality rate^d^	0.055	0.50	0.105	0.20	0.104	0.20
Physical environment						
Incidence rate^c^	0.106	0.19	0.163	*0.04*	0.008	0.93
Mortality rate^d^	0.060	0.46	0.184	*0.02*	0.091	0.26
Clinical care						
Incidence rate^c^	−0.134	0.10	0.001	0.99	0.118	0.14
Mortality rate^d^	−0.132	0.10	0.034	0.68	0.115	0.16

*ρ*, Pearson correlation coefficient. Italics indicate significant correlation (*P* < 0.05).

a Data unavailable for Taliaferro, Webster, and Echols counties.

b Includes Caucasian, African America, American Indian/Alaska native, Asian or Pacific Islander, and unknown race.

c Lincoln and Treutlen counties were identified as outliers and excluded from correlation analysis.

d Lincoln, Treutlen, Heard, and Wilkinson counties were identified as outliers and excluded from correlation analysis.

## Discussion

Our study is the first to examine the geographic distribution of pancreatic cancer by racial subgroup at the county level. We had a considerably large sample size of 10,877 pancreatic cancer cases and 9675 pancreatic cancer deaths, and pancreatic cancer incidence, mortality, and MIRs were examined from 2000 to 2011 among African Americans, Caucasians, and the entire Georgia population. Cluster analyses were conducted to identify unusual aggregations of cancer cases and deaths, which is valuable as recognizing areas of greater cancer risk can assist in efforts to prevent future disease development.

Our analyses suggest that pancreatic cancer racial disparities were present in Georgia. African Americans displayed an overall significantly greater age‐adjusted incidence (14.6/100,000) and mortality rate (13.3/100,000) compared to Caucasians (10.8/100,000 and 9.7/100,000). When examining annual disease rates, African Americans perennially had higher age‐adjusted incidence and mortality rates. Additionally, in terms of disease incidence and mortality by sex, African American males had the highest overall age‐adjusted incidence rate (16.5/100,000) and mortality rate (15.2/100,000). These findings align with previous publications and national trends 33. In the United States, African Americans have remained disproportionally affected by pancreatic cancer than Caucasians 33 and have the poorest median survival time compared to other races 23.

Although the disparity in pancreatic cancer incidence among African Americans is not fully understood, excess incidence among this racial subgroup may be related to lifestyle factors, such as increased cigarette smoking, alcohol use, and poor diet. In our study, we detected moderate nonsignificant positive correlations (*P* ≤ 0.10) between health behaviors and pancreatic cancer incidence and mortality rates for African Americans but not for rates for all races nor Caucasians. While these associations were not statistically significant, there are marked inconsistencies between the race‐specific rates and associations with health behaviors. The health behaviors measure used in this investigation was a composite measure which took into consideration multiple county‐level health behaviors including smoking, obesity, excessive drinking, motor vehicle crash deaths, sexually transmitted infections, and teen births. Based on previous investigations, some of these health behaviors may play a role in cancer development by race. For instance, a prior epidemiological investigation determined that excess pancreatic cancer incidence among African Americans relative to Caucasians could be explained by disease risk factors such as cigarette smoking and diabetes among men and heavy alcohol drinking and elevated body mass index among women 34. An additional review speculated that observed differences in pancreatic cancer incidence between African Americans and Caucasians could be related to differences in smoking habits, diabetes, body mass index, and vitamin D insufficiency 5.

Furthermore, lifestyle behaviors are regularly considered as major contributors to cancer etiology and distribution, including pancreatic cancer 35. A recent systematic review examined publically available state and federal cancer cluster investigation reports generated over the past 20 years and concluded that observed geographic disparities in cancer incidence are multifactorial and likely attributable to differences in risk factors such as cigarette smoking, poor diet, physical inactivity, obesity, and health care seeking behaviors 36. Accordingly, the geographic location of the clusters within this study may also be related to health behaviors within Georgia populations. In fact, Southwest and Central Georgia displayed the overall lowest health behavior rankings in the state in 2011 according to *County Health Rankings & Roadmaps* 29. Interestingly, disease clusters were found overlapping these “poor” health behavior ranking counties, suggesting that additional investigations should focus on examining health behaviors as they relate to pancreatic cancer development in Georgia.

In our study, six disease clusters were identified with a significantly greater than expected number of pancreatic cancer cases, and six clusters were identified with a significantly greater than expected number of pancreatic cancer deaths for the entire Georgia population. In particular, the southwest corner of Georgia and the central region of Georgia appeared to be areas of concern for both races; incidence or mortality clusters were present for both races within these regions of the state. Among African Americans, clustering appears to be focused in Georgia's Upper Coastal Plain, where agriculture focused in peanuts, cotton, and pecans is the major industry. Among Caucasians, a distinct cluster was identified in the northeast, in the Georgia Blue Ridge Mountains region, where the population is mostly Caucasian, rural, and has a median income below the State's.

In addition to lifestyle behaviors, the disease clustering in Georgia may be attributable, at least in part, to physical environment. In 2011, Southwest and central Northeast Georgia displayed some of the lowest physical environment rankings in the state according to *County Health Rankings & Roadmaps* 29. We identified disease clusters in some of the same counties identified as having a “poor” physical environment. Furthermore, weak but significant correlations were noted between physical environment and pancreatic cancer incidence and mortality rate among African Americans yet not Caucasians. The physical environment measure used in this investigation considered air pollution‐particulate matter days, air pollution‐ozone days, access to healthy foods, and access to recreational facilities. While this measure is composed of specific factors, it may be indicative of other environmental factors, such as water, air, ground, or industrial pollutants.

Investigations of relationships between potential environmental exposures and pancreatic cancer development should be explored in Georgia, as increased cancer risk has been associated with environmental factors in previous studies in the southeastern United States. For example, a 2011 investigation in South Carolina found uranium to be associated with an elevated risk for colorectal, prostate, and total cancer 37. In terms of disease development by race, other research examining environmental factors and county‐level cancer rates in GA has found inconsistent associations differing by county population race demographics 38. Similar findings were noted in our study, as correlations between physical environment and pancreatic cancer were only displayed among African Americans. Additionally, occupational exposures may play a role in cancer development as studies have found that occupational exposures, specifically chlorinated hydrocarbon compounds, could be associated with an elevated pancreatic cancer risk 39. In Georgia, the development of pancreatic cancer clusters by race may be associated with physical environment factors, such as water quality or industry. Further investigations are recommended.

As previously described, pancreatic cancer is a disease of notable mortality. Ultimately, the high MIRs identified within this study provided a depiction of the survival experience in Georgia and confirmed that pancreatic cancer was a tremendously fatal disease for all races in the state. Moreover, using national data, it is evident that pancreatic cancer is a severe disease relative to other cancer sites. In this study, the state MIR for pancreatic cancer (0.91) was comparable to the national MIR for pancreatic cancer (0.88) 1. It is also noteworthy that the national MIR of pancreatic cancer (0.91) is considerably higher than the national MIR of other cancer sites such as, breast (0.18), prostate (0.16), cervical (0.30), bladder (0.22), and colon and rectum (0.37) 1.

Previous investigations have noted racial differences in cancer MIRs in the United States 26, 27. One prior study examined racial cancer disparities in Georgia and determined that African Americans had higher MIRs than Caucasians for each cancer site (colorectal, prostate, breast, oral, and cervical) examined 26. Similar findings were also noted in South Carolina 27. However, no significant differences in pancreatic cancer MIRs were exhibited between African American and Caucasians in this study; county MIRs were frequently high (>0.89) for both African Americans and Caucasians across the majority of Georgia. The high MIR of pancreatic cancer for all races in Georgia illustrates the magnitude of pancreatic cancer severity and demonstrates the necessity of understanding disease etiology and improving disease prevention and treatment efforts among individuals of all racial backgrounds.

National organizations currently exist to reduce the incidence of pancreatic cancer and improve treatment options. The Pancreatic Cancer Action Network, a national organization dedicated to advancing research and supporting pancreatic cancer patients, believes that a comprehensive strategy is needed to make a significant impact on pancreatic cancer survival 40. This entails advancing research, building and sustaining federal support, providing information and education to patients and families, and raising disease awareness 40. Additionally, the National Pancreas Foundation, a nonprofit organization established to support the funding of research related to pancreatic diseases, provides hope for those suffering from pancreatic disease through funding innovative research, advocating for new and better therapies, and providing support and education for patients, caregivers, and health care professionals 41. Moving forward in disease prevention and management efforts, a comprehensive approach, such as that recommended by national organizations, is suggested for Georgia, especially in high‐risk areas such as the southwest and central region of the state.

Although we examined a large, high quality, and representative cancer registry with data spanning 11 years, several limitations of our study are acknowledged. Despite including 10,877 cases and 9675 deaths in our study, only 3082 of the cases and 2734 deaths occurred in African Americans. In fact, several counties (*n* = 17) had no reported cases of pancreatic cancer among African Americans. Of these counties, 13 had an African American population percentage of <5%. The lack of data for African Americans in these counties resulted in unavailable calculations for disease rates and MIRs, and may have been influential in the limited number of clusters exhibited among African Americans.

Additionally, several significant county clusters of incidence and mortality border other surrounding states. These clusters may be subject to an edge effect bias that ignores cross‐boundary influences. Shape effect may have also influenced disease clustering in Georgia. An additional concern is experiment wide error that is ignored in the SaTScan program's identification of secondary clusters. While our analysis attempts to address this limitation by setting a more conservative *P*‐value cut‐off point (*P* ≤ 0.01), interpretations of secondary clusters is limited.

Additionally, our study examined county‐level clusters rather than individual‐level clusters. Consequently, our investigation is limited in its ability to identify individual‐level clustering and may have overlooked intracounty clustering. Furthermore, the health factors we examined within our study were composite characteristics and do not solely represent specific risk factors of pancreatic cancer. These limitations should be kept in mind when considering the results of this study.

The implications of this study suggest that African Americans experience a disproportionate burden of pancreatic cancer incidence and mortality in Georgia, particularly in Georgia's Upper Coastal Plain. Additional research is warranted for all racial subgroups within Georgia, but special attention should be given to the state's African American population with a specific goal of exploring health behaviors, such as cigarette smoking and diet, and physical environment characteristics, such as water quality and potential occupational exposures. Cluster models suggested that the central and southwest region of Georgia exhibit significant pancreatic cancer clusters for both races. Ultimately, it is recommended that interventions, research, and health promotion strategies be implemented in these high‐risk areas. Understanding variations in disease clustering between Caucasians and African Americans will require a more thorough understanding of county demographics, environmental factors, and risk factors by race.

## Conflict of Interest

The authors declare that they have no conflict of interest.
